# A Novel Approach to Predict Sudden Cardiac Death (SCD) Using Nonlinear and Time-Frequency Analyses from HRV Signals

**DOI:** 10.1371/journal.pone.0081896

**Published:** 2014-02-04

**Authors:** Elias Ebrahimzadeh, Mohammad Pooyan, Ahmad Bijar

**Affiliations:** Department of Biomedical Engineering, Shahed University, Tehran, Iran; University of Minnesota, United States of America

## Abstract

Investigations show that millions of people all around the world die as the result of sudden cardiac death (SCD). These deaths can be reduced by using medical equipment, such as defibrillators, after detection. We need to propose suitable ways to assist doctors to predict sudden cardiac death with a high level of accuracy. To do this, Linear, Time-Frequency (TF) and Nonlinear features have been extracted from HRV of ECG signal. Finally, healthy people and people at risk of SCD are classified by k-Nearest Neighbor (k-NN) and Multilayer Perceptron Neural Network (MLP). To evaluate, we have compared the classification rates for both separate and combined Nonlinear and TF features. The results show that HRV signals have special features in the vicinity of the occurrence of SCD that have the ability to distinguish between patients prone to SCD and normal people. We found that the combination of Time-Frequency and Nonlinear features have a better ability to achieve higher accuracy. The experimental results show that the combination of features can predict SCD by the accuracy of 99.73%, 96.52%, 90.37% and 83.96% for the first, second, third and forth one-minute intervals, respectively, before SCD occurrence.

## Introduction

Sudden cardiac death is natural death from cardiac causes, heralded by abrupt loss of consciousness within one hour of the onset of acute symptoms [1]. This is a very serious cardiac event that can deprive patient’s life within several minutes [2]. Despite the significant decline in coronary artery disease (CAD) mortality in the second half of the 20th century [3], sudden cardiac death (SCD) continues to claim 250 000 to 300 000 US lives annually [4]. In North America and Europe the annual incidence of SCD ranges between 50 to 100 per 100 000 in the general population [5]–[8]. Because of the absence of emergency medical response systems in most world regions, worldwide estimates are currently not available [9]. However, even in the presence of advanced first responder systems for resuscitation of out-of-hospital cardiac arrest, the overall survival rate in a recent North American analysis was 4.6% [10], [11]. Astonishingly, the victim may not even have been diagnosed with heart disease. Also, the time and mode of death happen unexpectedly [12]. Most victims (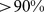) have previously known or unrecognized cardiac abnormality [13]–[17].

The most common cause of sudden cardiac death in adults over the age of 30 is coronary artery atheroma. The most common finding at postmortem examination is chronic high-grade stenosis of at least one segment of a major coronary artery, the arteries which supply the heart muscle with its blood supply. A significant number of cases also have an identifiable thrombus (clot) in a major coronary artery which causes transmural occlusion of that vessel. Left ventricular hypertrophy is the second leading cause of sudden cardiac death in the adult population. This is most commonly the result of longstanding high blood pressure which has caused secondary damage to the wall of the main pumping chamber of the heart, the left ventricle. Hypertrophy, as well, is associated with cardiac arrhythmias. The mechanism of death in the majority of patients dying of sudden cardiac death is ventricular fibrillation; as a consequence, there may be no prodromal symptoms associated with the death. Patients may be going about their daily business and suddenly collapse, without any typical features of myocardial infarction (heart attack) like chest pain or shortness of breath [18]. However, it may abruptly strike any person if he or she possesses of high risk heart disease, even young person, and athlete. Besides utilizing public access defibrillation (PAD) procedure to rescue impending death patient after collapse, the better way is to prevent onset SCD by adopting medical aid prior to collapse. Thus, is it possible to make an early warning, even before crisis presenting half an hour? [19] Ichimaru et al. found that the respiratory peak of the heart rate variability (HRV) in SCD patient was disappeared during the night time one-week before death [20]. Van Hoogenhuyze, D., Martin, et al. observed two HRV measurements, standard deviation of mean of sinus R-R intervals (SDANN) and mean of SD (SD), from 24 hrs HRV. They had evidenced to show that HRV is low in patients who experience SCD, and is high in young healthy subjects [21]. In our early and encouraging experiments, we showed that the TF method can classify normal and SCD subjects, more efficient than the classical method [22]. Moreover, we evaluated both TF and Classic methods by a MLP classifier for one minute ECG signal before SCD by an accuracy of 99.16% and 74.36%, respectively. However, the relationship between short-term HRV and SCD is unknown. In addition, repolarization alternans phenomena provides a safe, noninvasive marker for the risk of SCD, and has proven equally effective to an invasive and more expensive procedure - invasive electrophysiological study (EPS), which is commonly used by cardiac electrophysiologists [23], [24]. Analysis of heart rate variability (HRV) has provided a non invasive method for assessing cardiac autonomic control [25]. HRV is accepted as a strong and independent predictor of mortality after an acute myocardial infarction [26], such that a reduced HRV is associated with a higher risk for severe ventricular arrhythmia and sudden cardiac death [27]. In this article, the common Linear and Time-Frequency domain features, which have been extracted from heart rate variability (HRV) signal, are used to detect and predict sudden cardiac death (SCD). Although until now different Linear methods have been used for analysis of HRV signal, recently researches have shown that the Nonlinear processing methods gave more information than Linear methods and it was a good complement for them [28]. In addition, researches have shown that classic Linear methods do not have enough ability to predict the SCD [18], [22]. So the Nonlinear analysis is also done. In other words, in this research, we applied classic Linear, Time-Frequency and Nonlinear analysis on HRV signal of healthy persons and patients prone to SCD. Finally, by making use of a composition feature vector and a neural network classifier, it is possible to separate these two groups and predict the risk of SCD. Therefore, at first, Linear features are obtained from HRV signal which is extracted from ECG signal. Then, the Wigner Ville transform is applied to the HRV signal and thereupon time-frequency and Nonlinear features are extracted. At the next stage, feature selection is applied to reduce the number of features and a new combinational feature vector is proposed. Finally, K-Nearest Neighbor (k-NN) and Multilayer Perceptron (MLP) neural network are used to classify healthy persons and persons who are susceptible to heart death. To evaluate the performance of the proposed method in prediction of SCD, feature vectors are extracted from different segments of the signals (at successive intervals of 1 minutes) as below

1. The first interval before SCD.
2. The second interval before SCD.
3. The third interval before SCD.
4. The forth interval before SCD.

**Figure 1:**
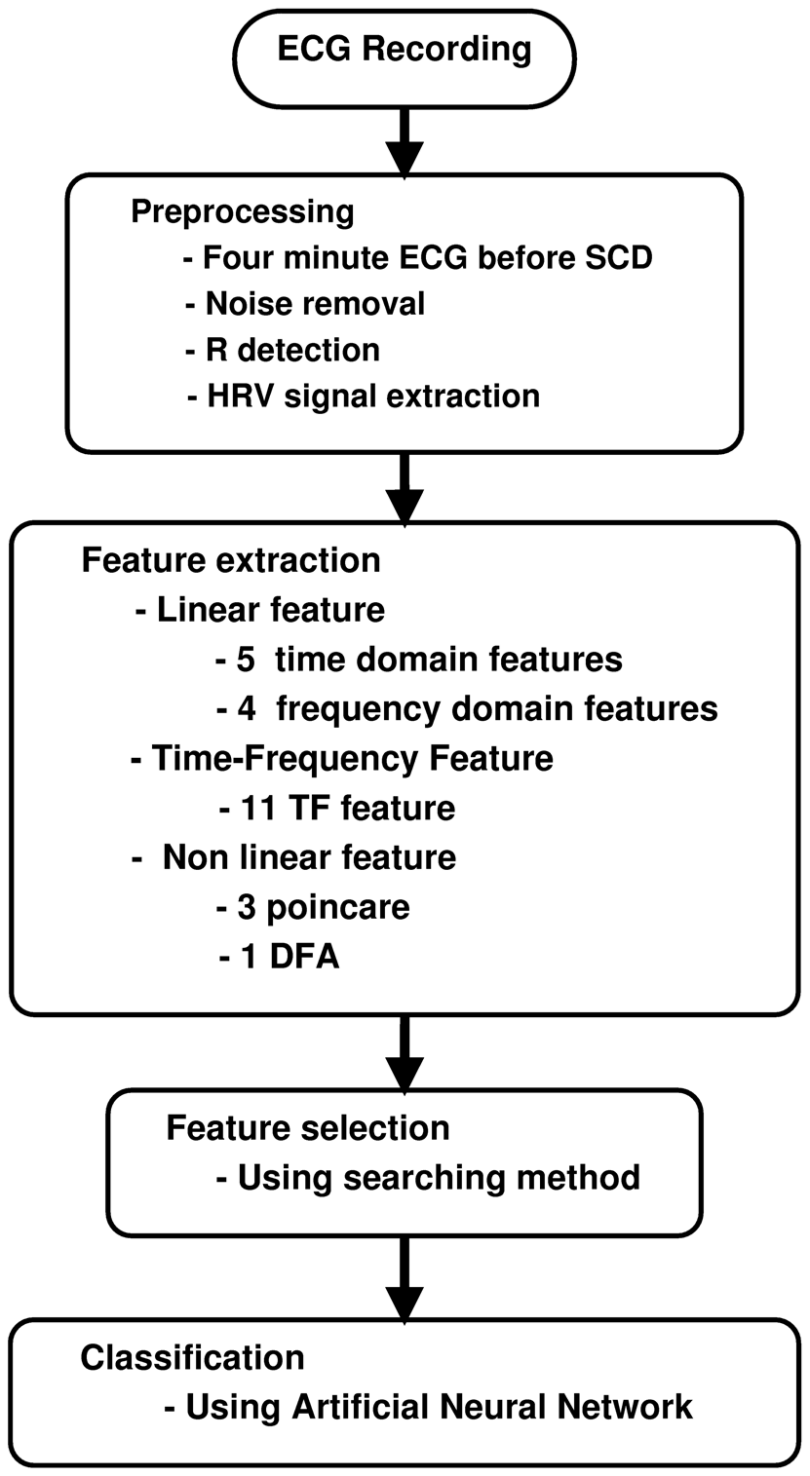
Block diagram of the proposed approach for prediction of SCD.

The capability of each one minute interval (i.e., the first one minute, the second one minute, the third one minute and the forth one minute before SCD) in prediction of SCD is evaluated through the computing of Separability factor. Based on the explanations mentioned above, the block diagram of our approach for prediction of SCD is shown in Figure 1.

## Materials and Methods

The proposed method is evaluated on a database containing ECG signals from 35 patients with sudden cardiac death (including 16 female and 19 male, aged 18 to 89, and with a sampling rate of 256 Hz) and 35 normal people (including 5 male, aged 26 to 45, and 13 female, aged 20 to 50, and with a sampling rate of 128 Hz). This open access database is prepared by MIT-BIH database with the title of Sudden Cardiac Death Holter database and Normal Sinus Rhythm database [29]. It is reminded that there were some patients with two channels of ECG signal where both of them were used as input observations, so the total number of signals are 70 for normal and patients.

### Preprocessing

**Figure 2:**
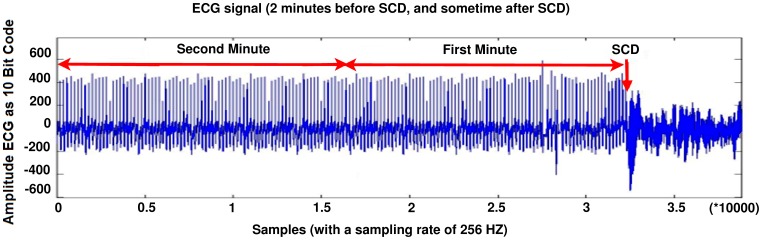
The ECG signal of SCD patient, from 2 minute before SCD event and several seconds after that.

The dataset consists of 24-hour ECG recordings (Holter) before the event of heart death and several seconds afterwards. Patients who showed signs of a previous heart attack or had the hard tachyarrhythmia were susceptible to SCD, and finally circum to SCD. The ECG signal (before SCD) of these patients is partitioned into one minute intervals (i.e., the first one minute, the second one minute, the third one minute and the forth one minute before SCD). Figure 2 shows an Electrocardiogram signal of a 34 years old patient that can lead to sudden cardiac death.

**Figure 3:**
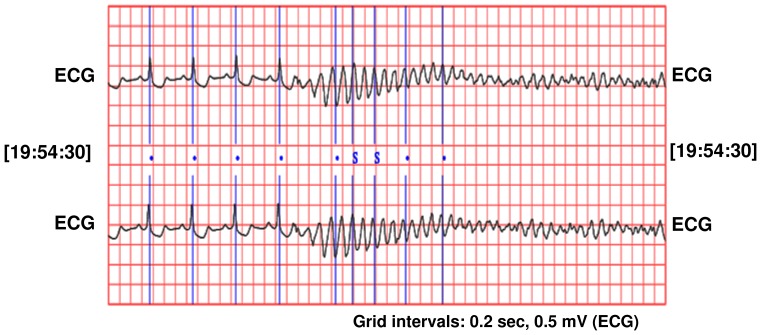
ECG signal of a person on the moment of heart death.

**Figure 4:**
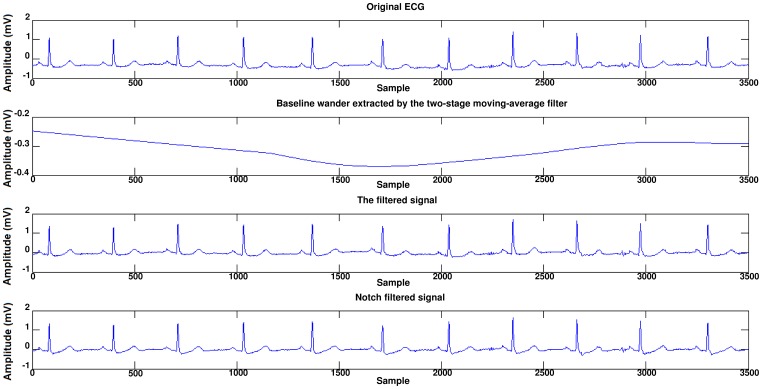
Noise reduction of a typical ECG signal.

**Figure 5:**
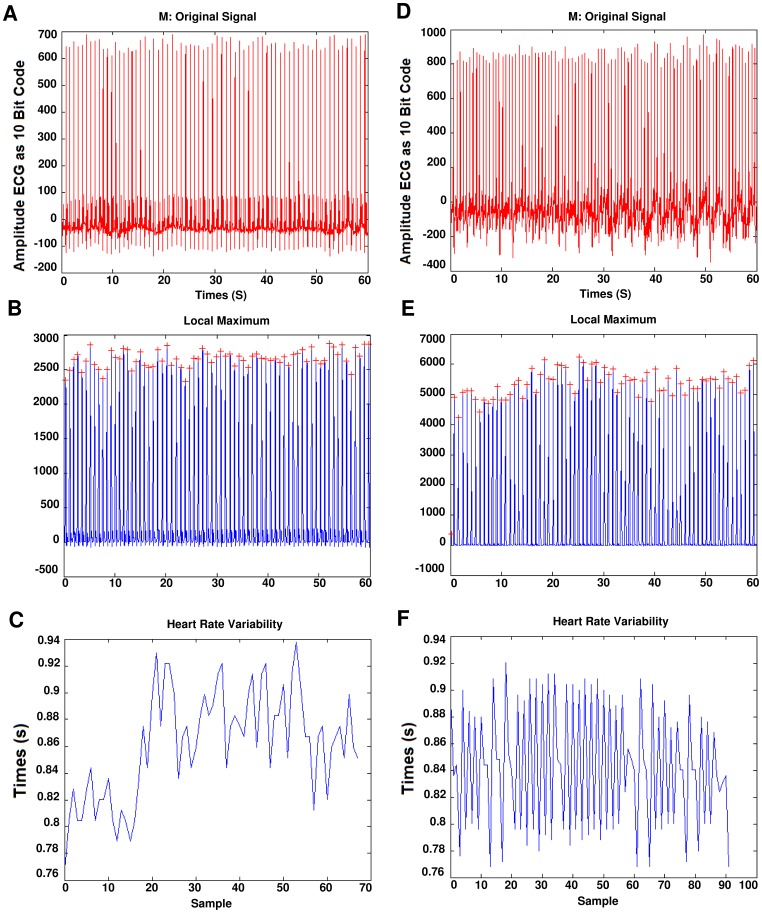
Extraction of HRV from ECG signal. (a) One minute of the ECG signal of a healthy person. (b) Extraction of QRS-complexes. (c) The HRV signal which was extracted from (a). (d) One minute the ECG signal of a patient just before occurrence of SCD. (e) Extraction of QRS-complexes. (f) The HRV signal which was extracted from (d).

Before occurring of SCD, there is no significant difference between the ECG signal for a person who is susceptible to heart death and the ECG signals of normal persons. In Figure 3, a sample of ECG signal of a person with SCD, several seconds before occurrence of SCD and a few seconds after it, is shown. The signal of the ECG of patient just one minute before the occurrence of the sudden cardiac death was selected as ECG recordings for patients. For normal subjects one minute of the ECG signal was selected at random. First of all, Noise reduction in ECG signals is done. Baseline wander due to respiration contains low frequency components and power line interference contains high frequency components. All ECG signals are filtered with moving-average filter to remove the baseline wander. In the moving-average filter [30], the first- and second-stage averaging window lengths are set to be 1/3 and 2/3 of the length of the input signal in samples, respectively. This filter is used to extract the baseline drift and place the output signal on the isoelectric line of the ECG recording. Then power-line frequency is removed from the median filtered ECG with a notch filter [31]. Figure 4 shows this process step by step. The filtered ECG signals are used in all subsequent processing. Then, the Pan-Tompkins [32] algorithm was used to detect the QRS-complexes in the ECG-signal from which we could determine the RR-intervals and HRV signal (R is a point corresponding to the peak of the QRS complex of the ECG wave; and *RR* is the interval between successive Rs. The term *NN* can be used in place of RR to emphasize the fact that the processed beats are *normal* beats). The preprocessed HRV signal is now ready to be extracted features from it. HRV and ECG signal of a healthy subject and a SCD one are shown in Figure 5.

## Classical Features Analysis

In this step some usual Linear features in time domain and frequency domain are extracted. These features, include 5 features in the time domain and 4 feature in the frequency domain.

### Time-domain feature

Statistical time-domain measures were divided into two classes:

- Direct measurements of RR intervals (or NN intervals)
- Measurements from the differences between RR intervals

#### Direct measurements of RR intervals:

These features include two simple time domain variables that can be calculated by

1. Mean of all RR intervals (MNN).
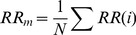
(1)


2. Standard deviation of all RR intervals (SDNN).
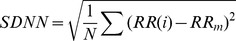
(2)


#### Measurements from the differences between RR intervals:

The square root of the mean of the squares of differences between adjacent RR Intervals (RMSSD).
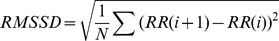
(3)


2. The standard deviation of differences between adjacent RR intervals (SDSD).
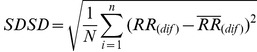
(4)

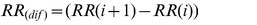
(5)

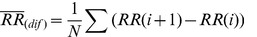
(6)


3. The proportion derived by dividing the number of interval differences of RR intervals greater than 50 ms by the total number of RR intervals (PNN50) [33].
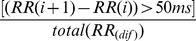
(7)


**Figure 6:**
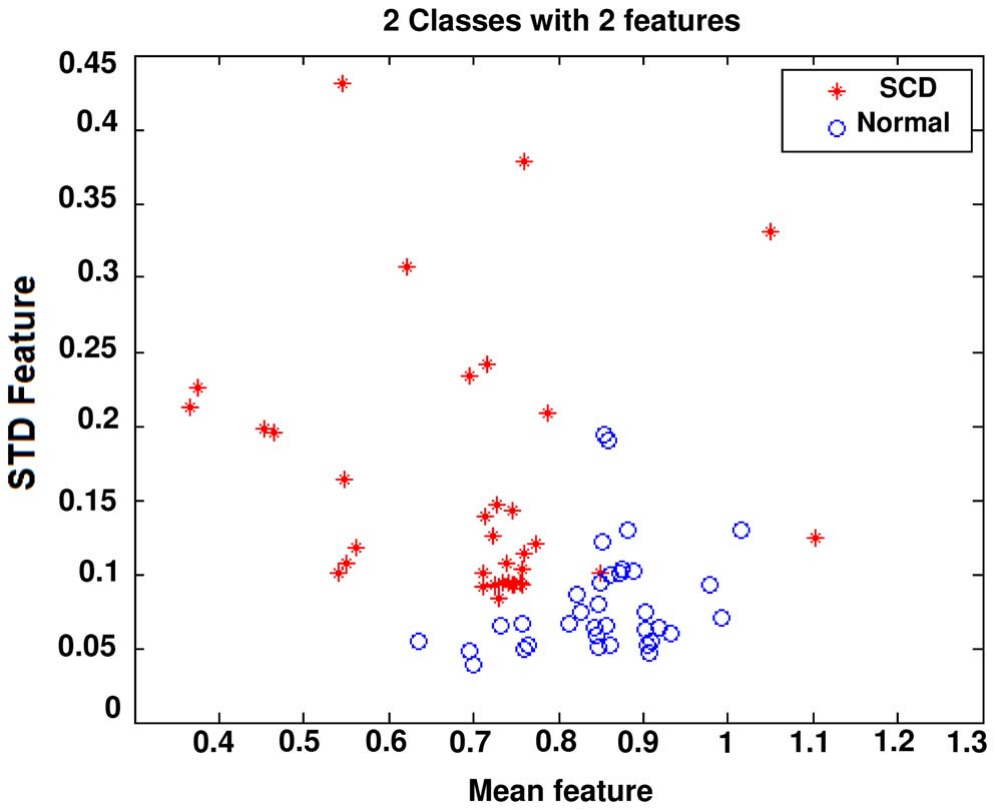
Spatial distribution of mean and STD features.

Spatial scattering of two of these features is shown in Figure 6. As seen in this Figure, theses features are suitable for discriminating between the two groups; healthy and SCD.

### Frequency domain features

**Figure 7:**
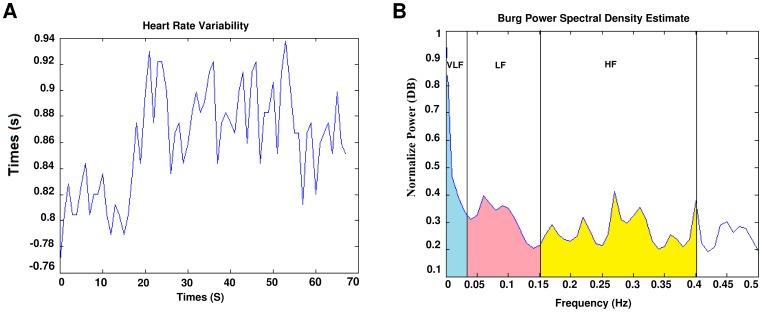
HRV signal and it’s power spectral density. (a) Extracted HRV signal. (b) PSD of HRV signal, power in each frequency band is indicated.

Although the time domain parameters are computationally effective but they lack the ability to discriminate between the sympathetic and parasympathetic contents of the RR intervals. It is generally accepted that the spectral power in the high frequency (HF) band (0.15–0.4 Hz) of the RR intervals reflects the respiratory sinus arrhythmia (RSA) and thus cardiac vagal activity. On the other hand, the low frequency (LF) band (0.04–0.15 Hz), is related to the baroreceptor control and is mediated by both vagal and sympathetic systems [26]. In this work, the LF,HF, and VLF (Very Low Frequency) bands PSD and ratio of the LF and HF bands power spectral density (LF/HF) are used as the frequency domain features of the RR interval signal [34]. The power spectral density(PSD) which is shown in Figure 7, was computed by Burg parametric method.

## Time-frequency Domain Analysis

**Figure 8:**
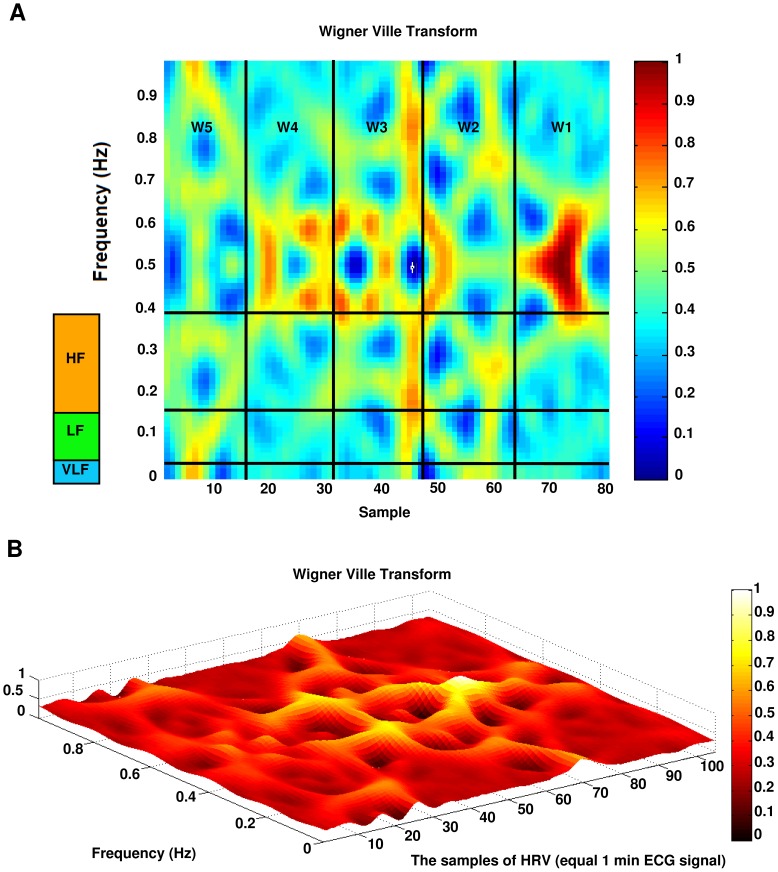
Wigner Ville transform of the HRV signal. (a) 2D view of a subject(b) 3D view of another subject.

Another approach to analyze non stationary HRV signal, is time-frequency (TF) method. This can be divided into three main categories: nonparametric linear TF methods based on linear filtering, including the short-time Fourier transform [35], [36] and the wavelet transform [37], [38], nonparametric quadratic TF representations, including the Wigner-Ville distribution and its filtered versions [39]–[43], and parametric time-varying methods based on autoregressive models with time-varying coefficients [44], [45]. In this paper the Smoothed Pseudo Wigner-Ville distribution (SPWVD) is preferred, since it provides better time frequency resolution than nonparametric linear methods, an independent control of time and frequency filtering, and power estimates at lower variance with parametric methods when rapid changes occur [40]. The main drawback of the SPWVD is the presence of cross-terms, which should be suppressed by the time and frequency filtering. The SPWVD of the discrete signal 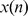 is defined by [41].
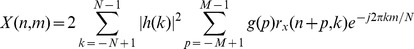
(8)where n and m are the discrete time and frequency indexes, respectively, h(k) is the frequency smoothing symmetric normed window of length 2N
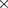
1, g(p) is the time smoothing symmetric normed window of length 2M
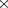
1 and 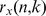 is the instantaneous autocorrelation function, defined as



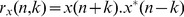
(9)
Figure 8 shows the result of applying Wigner Ville transform to the HRV signal.

### TF features extraction

Each HRV signal is divided into 5 segments of equal length, each segment is approximately 15 seconds in time domain. The average energy of each segment was computed. The features are:

**MAX w**: maximum amount of energy in each window.

**MIN w**: minimum amount of energy in each window.

**DIF w**: difference between maximum and minimum amount of energy between windows.

**STD w**: standard deviation between energy of time windows.

The obtained signal in TF domain is also divided into three frequency segments.

**E
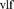
**: the total energy of signal in very low frequency band (0.003–0.04) Hz, divided by length of band (0.037).

**E
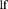
**: the total energy of signal in low frequency band (0.04–0.15) Hz, divided by length of band (0.11).

**E
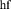
**: the total energy of signal in high frequency band (0.15–0.4) Hz, divided by length of band (0.25).

**F
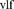
**: the average of energy signal in very low frequency band (0.04–0.003) Hz.

**F
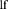
**: the average of energy signal in low frequency band (0.04–0.15) Hz.

**F
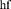
**: the average of energy signal in high frequency band (0.15–0.4) Hz.

Also, we have defined the *first order derivative* as a feature to show the difference between adjacent windows. This derivative is the difference between the average energy in subsequent windows. This derivative for the first window(first 15 S) was computed by the difference between this window and the last 15 seconds in the second minute. So the *first order derivative* feature is computed as below
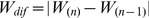
(10)


The result of features survey in time span of 15 seconds illustrates that in SCD person, the features changes from one window to next window is much more dominant so that we define the first order derivative.

## Nonlinear Analysis

Considering that cardiovascular system has Non-stationary behaviors and also is more complex than a Linear system, two Nonlinear analyses are used to illustrate chaotic dynamical characteristics in HRV signal in addition to the time-frequency features. In this way, four different Nonlinear parameters of the RR intervals are extracted in this work, which are described as below.

### Poincaré plot

**Figure 9:**
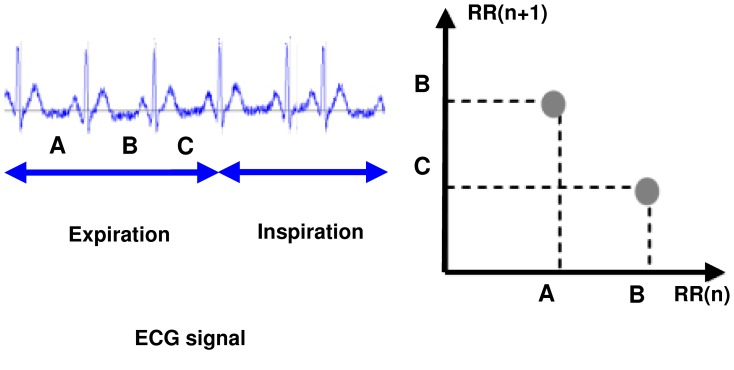
Poincaré plot.

**Figure 10:**
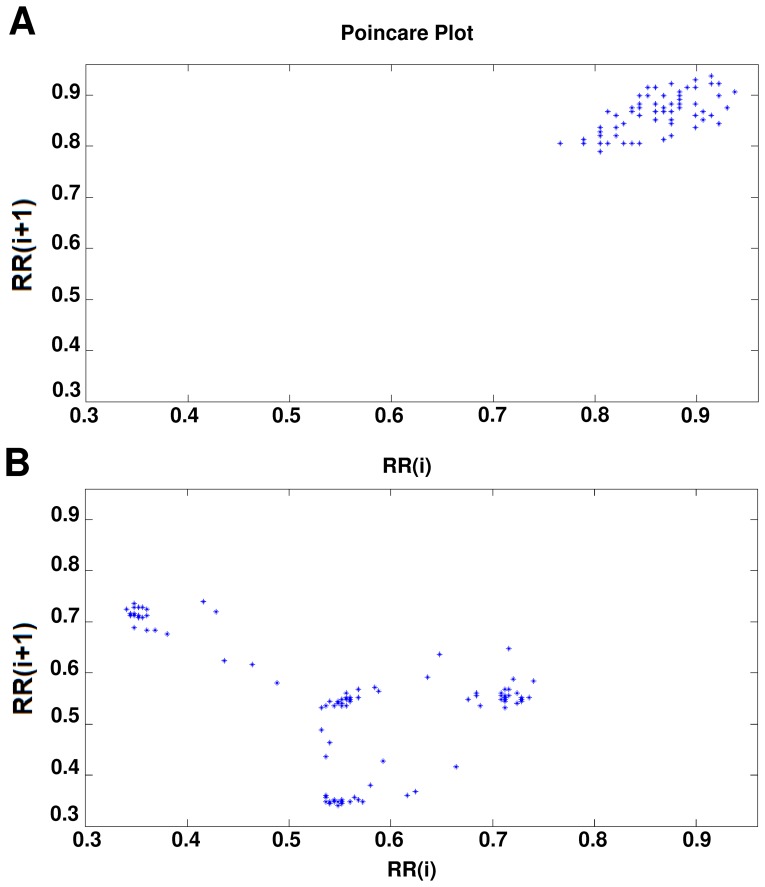
Poincaré plot. (a) Normal persons. (b) SCD Persons.

When in the RR intervals, each interval 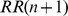 is plotted as a function of previous interval 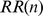, the resulting plot is known as the Poincaré plot. Poincaré plot can be seen as a graphical representation of the correlation between the successive RR intervals. This plot can be quantitatively analyzed by calculating the standard deviations of the distances of the points 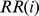 from the lines y = x and y = −x +2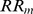, where 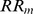 is the mean of all RR(i) values. The computation procedure of Poincaré plot is shown in Figure 9. These standard deviations are denoted by SD1 and SD2, respectively. In fact, SD 1represents the fast beat-to-beat variability, while SD2 describes the relatively long-term variability in the HRV signal [46]. The length (SD2) and the width (SD1) of the long and short axes of Poincaré plot images represent short and long-term variability of any Nonlinear dynamic system [47]. We developed mathematical formulations that relate each measure derived from Poincaré plot geometry in order to well understanding existing heart rate variability indexes [47]. A strong correlation was found when comparing high frequency power of heart rate signals (modulated by parasympathetic nervous system) with SD1 [48]. SD2 was found to be well correlated with both low and high frequency power (modulated by both the parasympathetic and sympathetic nervous system) [48]. The ratio SD1/SD2 is usually used to describe the relation between the two components [33], [49], [50]. Figure 10 shows the Poincaré plot of normal person and SCD persons.

### DFA analysis method

Detrended fluctuation analysis (DFA) is a method for quantifying long-range correlations embedded in a seemingly non-stationary time series, and also avoids the spurious detection of apparent long range correlations that are artifacts of non stationarity. This method is a modified root mean square analysis of a random walk [33], [51]–[56].

## Feature Selection

Using the obtained features from Linear, Time-Frequency and Nonlinear Processings and constituting a new combinational feature vector brings a good separability between two classes (i.e., Healthy people and People at risk of SCD). But in any classification task, there is a possibility that some of the extracted features might be redundant. These features can increase the cost and running time of the system, and decrease its generalization performance. In this way, the selection of the best discriminative features plays an important role when constructing classifiers. To identify the best features (for classification) in feature space, searching selection method is applied. In such way, first the classification has been applied separately to each feature. The best feature has been selected in accordance with the most value of classification accuracy. This feature will be combined with the other individual features and thus the best pair combination will be produced. The optimal feature space is achieved when the minimum number of features results in the highest classification accuracy (i.e., when adding a new feature does not increase the classification accuracy). So, this process is stopped when adding a new feature decreases the classification accuracy or does not result in an increase in it. It is reminded that in each step the training process is done only using the train data and after performing the feature selection and afterwards determining the best combination of features, the session of testing is done using the remained sample. Considering that there are 70 samples, this procedure is repeated 70 times, in each step 69 samples are used as train data to determine the best combination of features and the remained one is considered as test data. In other words, each time this process is run, a different optimal combination of features is obtained using a different train and test data. The obtained best features for a sample step of this process are listed in Table 1.

**Table 1:** Comparison of the thirteen extracted HRV parameters from control, and SCD dataset.

Parameters	Normal dataset (n = 35)	SCD dataset (n = 34)	p-Value
	Mean±SD	Mean±SD	
Mean NN (ms)	876±134	706±147	<0.001
SDNN (ms)	58.4±3.9	64.2 ± 4.3	0.56
RMSSD (ms)	42.1±4.2	54.6±3.2	0.13
pNN50 (%)	10.4±13.3	7.28± 10.5	0.07
VLF (ms^2^)	20.6±64.3	27.9± 85.2	0.63
HF (ms^2^)	570±139	602 ± 1019	0.79
LF/HF	0.75±0.57	0.75± 0.99	0.89
SD_1_ (ms)	24.5±24.5	27.8±26.7	0.53
SD_1_/SD_2_	0.32±0.19	0.36±0.22	0.31
*α*(*DFA*)	0.83±0.15	1.12±0.18	0.45
DIFw(TF)	19.65±3.17	42.73±12.76	0.29
STDw(TF)	8.03±2.34	23.37±7.08	0.33
W*_dif_*(*TF*)	39.14±4.15	80.03±17.29	0.26

## Classification

To discriminate between ECG of normal person and a person who is prone to sudden cardiac death, the Multilayer perceptron (MLP) neural network and K-Nearest Neighbor (k-NN) classifier have been used. Features extracted from HRVs of one minute intervals (i.e., the first one minute, the second one minute, the third one minute and the forth one minute before SCD) were compared with normal HRVs of one minute.

### Multilayer perceptron neural network

The classifier using a three-layer MLP with error back propagation algorithm and variable learning rate. The input layer has a number of nodes equal to the input vector length (13 node). The output layer consists of one node, accounting for a possibility of only 2 classes to be classified. Also, All the possible combinations of the selected numbers of neurons in the hidden layer were selected and trained and finally the optimized number was equal to 5. The output nodes had Linear transfer functions, and the hidden layer used a sigmoid function. Network training continued until the mean square error became less than 0.01 or the number of training iterations reached to 1000. Due to the limited input data set, Leave One Out cross-validation method was done for training [56]. At each stage one of observations was selected as test data and 69 as train data, and this process repeated 70 times. Network error in each step was computed, and finally the average was calculated. This process was done for 16 times to reach the average accuracy. One advantage of this approach is that all the input data set are represented in both processes (train and test).

### k-Nearest neighbor

The k-nearest neighbor algorithm (k-NN) is a non-parametric method for classifying objects based on closest training examples in the feature space [57]. k-NN is a type of instance-based learning where the function is only approximated locally and all computation is deferred until classification. The k-nearest neighbor algorithm is amongst the simplest of all machine learning algorithms: an object is classified by a majority vote of its neighbors, with the object being assigned to the class most common amongst its k nearest neighbors (k is a positive integer, typically small). Over several distance measures that might be used in this algorithm, Euclidean distance is commonly preferred as the distance measure. If k = 1, then the object is simply assigned to the class of its nearest neighbor. The selected feature set is then used to determine the best value of k for the classifier. Therefore, different numbers of nearest neighbors (k = 1, 3, 5, 7, 9) are tested in the k-NN classifier to obtain the best performance for the classifier [58]. Performances of all classifiers are calculated based on their accuracy. The maximum performance is provided by a 7-nearest neighbor classifier.

## Evaluation

The ability of the proposed method for prediction of sudden cardiac death is evaluated using accuracy (AC), sensitivity (SN), specifity (SP) and precision (P). In the following Equations (4)(10), TP refers to true positives (correctly predicted SCD), TN refers to true negatives (correctly predicted non-SCD), FN refers to false negatives (incorrectly predicted non-SCD) and FP refers to false positives (incorrectly predicted SCD).

**Accuracy** (AC): proportion of correct predictions to the total predictions
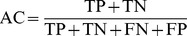
(11)


**Sensitivity** (SN): proportion of true positives to the total positives
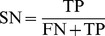
(12)


**Specificity** (SP): proportion of true negatives to the total negatives
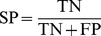
(13)


**Precision** (P): proportion of predicted positive cases that were correct
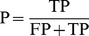
(14)


To evaluate the proposed method, AC, SN, SP and P are computed for MLP classifier for one, two, three and four minutes before SCD (to evaluate the robustness of the system, this procedure is repeated for 16 times).

## Results

After selecting the classifier, in order to evaluate the separability of features, the extracted features are compared with each other in both individual (Linear, Nonlinear and Time-Frequency) and optimal combinational mode. The separability of Linear, Nonlinear and Time-Frequency features and also combinational mode for three minutes (180 s) and two minutes(120 s) before SCD are shown in Table 2.

**Table 2:** The accuracy of MLP and k-NN classifiers with the selected features subsets (individual and combinational) for Healthy and patients prone to SCD.

Average Classification Rate				
Features	Two-Minutes (120 S before SCD)		Three-Minutes(180 S before SCD)	
	MLP	k-NN	MLP	k-NN
Linear	71.08%	69.57%	68.82%	68.13%
Time-Frequency	80.16%	77.13%	76.41%	74.96%
Nonlinear	85.38%	84.33%	82.17%	83.58%
combinational	98.74%	96.42%	95.78%	93.63%

**Figure 11:**
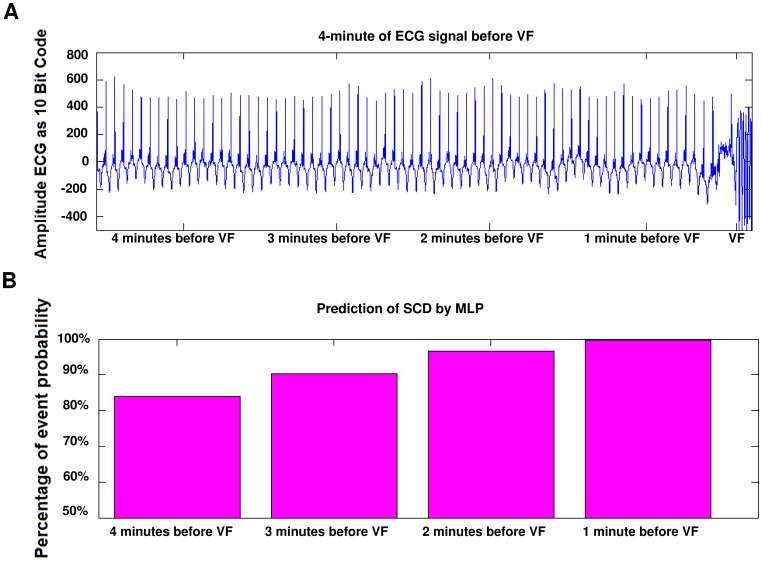
Prediction of SCD by classification accuracy 4 minutes before ventricular failure (VF). (a) ECG signal of a patient prone to SCD. (b) Prediction of SCD by computing the percentage of event probability.

As can be seen in Table 2, combinational features have more capability in classification of people (i.e., Normal and SCD). So that’s why combinational features have been used in this study as input features vector to predict SCD. For this goal, HRV signals (before SCD) have been partitioned into one minute intervals. Then, the separability of each one minute interval (i.e., the first one minute, the second one minute, the third one minute and the forth one minute before SCD) in prediction of SCD is evaluated by computing the accuracy. The obtained results show that the combinational feature vector can predict SCD by the accuracy of 99.73%, 96.52%, 90.37% and 83.96% for the first, second, third and forth one minute intervals, respectively. The results show that the two minutes interval before SCD contains more information related to the SCD which can be used for prediction. In addition, the first one minute interval before SCD contains much more precious information for prediction of SCD in comparison with other intervals (i.e., the second, third and forth intervals), which is expectable from the medical perspective. To have a reasonable comparison with different intervals, the performance of MLP classifier is computed using AC, SN, SP, and P measures for all intervals (this process is repeated for sixteen times). The obtained results are reported in Tables 3 to 7. Also, the ability of combinational features vector in predicting of SCD is evaluated through the k-NN classifier by the accuracy of 98.32%, 95.04%, 88.93% and 81.49%. Table 8 shows average separating percent,4 minutes before incident. To give more understanding, ECG recording of a patient who experienced SCD during the recording and the percentages of SCD prediction through the proposed method are shown in Figure 11.

**Table 3:** Run the program 16 times for *First one minute* by means of MLP classifier and composition feature vector.

Time	1	2	3	4	5	6	7	8	9	10	11	12	13	14	15	16	Average
TP	35	35	35	**34**	35	35	**34**	35	35	35	35	35	35	35	35	35	34.875
TN	35	35	35	35	35	35	35	35	35	35	35	35	35	35	35	**34**	34.938
FP	0	0	0	0	0	0	0	0	0	0	0	0	0	0	0	**1**	0.062
FN	0	0	0	**1**	0	0	**1**	0	0	0	0	0	0	0	0	0	0.125

TP: True Positive, TN: True Negative, FP: False Positive, FN: False Negative.

**Table 4:** Run the program 16 times for *Second one minute* by means of MLP classifier and composition feature vector.

Time	1	2	3	4	5	6	7	8	9	10	11	12	13	14	15	16	Average
TP	34	35	35	34	35	32	34	34	33	34	33	34	35	33	33	34	33.875
TN	33	33	35	34	35	34	34	32	33	34	35	33	33	34	33	34	33.687
FP	2	2	0	1	0	1	1	3	2	1	0	2	2	1	2	1	1.312
FN	1	0	0	1	0	3	1	1	2	1	2	1	0	2	2	1	1.125

TP: True Positive, TN: True Negative, FP: False Positive, FN: False Negative.

**Table 5:** Run the program 16 times for *Third one minute* by means of MLP classifier and composition feature vector.

Time	1	2	3	4	5	6	7	8	9	10	11	12	13	14	15	16	Average
TP	32	31	32	33	31	32	33	33	31	32	30	28	31	31	32	30	31.375
TN	33	33	32	30	32	31	31	32	32	31	32	33	31	32	32	33	31.875
FP	2	2	3	5	3	4	4	3	3	4	3	2	4	3	3	2	3.125
FN	3	4	3	2	4	3	2	2	4	3	5	7	4	4	3	5	3.625

TP: True Positive, TN: True Negative, FP: False Positive, FN: False Negative.

**Table 6:** Run the program 16 times for *Forth one minute* by means of MLP classifier and composition feature vector.

Time	1	2	3	4	5	6	7	8	9	10	11	12	13	14	15	16	Average
TP	31	31	28	30	29	30	30	29	29	31	25	28	27	30	32	29	29.313
TN	28	30	30	30	30	31	28	29	27	27	32	31	31	29	28	30	29.437
FP	7	5	5	5	5	4	7	6	8	8	3	4	4	6	7	5	5.563
FN	4	4	7	5	6	5	5	6	6	4	10	7	8	5	3	6	5.687

TP: True Positive, TN: True Negative, FP: False Positive, FN: False Negative.

**Table 7:** Accuracy, Sensitivity, Specificity, and Precision measures for all intervals before SCD.

Method	Accuracy	Sensitivity	Specificity	Precision
One minute beforeSCD	**99.73%**	**99.64%**	**0.001%**	**99.82%**
Two minutes beforeSCD	96.52%	96.78%	0.037%	96.27%
Three minutes beforeSCD	90.36%	89.64%	0.089%	90.94%
Four minutes beforeSCD	83.93%	83.75%	0.159%	84.04%

**Table 8:** Average of separating percent between healthy person and patients prone to SCD, 4minute before incident, by means of composition vector motion method.

Average Classification Rate with composition feature vector				
Classifier	Forth one minute	Third one minute	Second one minute	First one minute
MLP	83.96%	90.37%	96.52%	99.73%
k-NN	81.49%	88.93%	95.04%	98.32%

Although there is not a significant difference between normal ECG and those patients which prone to SCD, by using the proposed combinational feature vector, symptoms of SCD can be observed even 4 minutes before SCD. In other words, in spite of that Cardiology and Electrocardiography Experts cannot distinguish between normal ECG and patients which prone to SCD, the proposed extracted features can be used to predict SCD. It is reminded that those intervals which are closer to SCD have more capability for prediction of SCD.

## Discussion

In this paper a new approach, for prediction of sudden cardiac death, is proposed. After extraction of the HRV signal from ECG signal, some Linear, Time-Frequency (TF) and Nonlinear features have been extracted from HRV signal. Then, the dimension of features space is reduced by applying feature selection and finally, healthy people and people at risk of SCD, are classified by k-NN (k Nearest Neighbor) and MLP (Multilayer Perceptron) neural network.

To evaluate the capabilities of analytical methods in classification, we have compared the classification rates for both separate and combined Nonlinear and TF features. These results are compared with the previous results, reported by other researcher such as Shen et al. [18] which used similar methods of evaluation. Shen et al. [18] developed a personal cardiac homecare system by sensing Lead-I ECG signals for detecting and predicting SCD events. The wavelet analysis was applied to detect SCD and the overall performance was 87.5% correct detection rate. In addition, artificial neural networks (ANN) were used to predict SCD events. The correct prediction rates by applying least mean square (LMS), decision based on neural network (DBNN), and back propagation (BP) neural network were 67.44%, 58.14% and 55.81%, respectively. For the SCD detection, their own database with 20 normal sinus rhythms and the MIT/BIH SCD database with 20 subjects without ventricular failure (VF) onset were combined as detection training data set. For the SCD prediction, again, their own database with 20 normal sinus rhythms and total of 23 SCD samples built their prediction training data set. The 23 SCD samples were obtained as follows: Two-minute ECG signals before VF onset times were kept in their SCD database in order to simulate two-minute right before SCD. For the testing dataset, forty randomly selected subjects from campus were studied.

To compare our approach with the method proposed by Shen et al., we simulated and evaluated their method with MIT/BIH SCD database. Therefore comparison of our method with their method is reasonable. This comparison is done in Table 9. As it is seen, the predictive accuracy has been improved from 67.44% to 98.74%.

**Table 9:** Predictive accuracy for the proposed method and Shen’s method [18] (2-minute analysis).

Classifier	Method		
	Shen *et al.*	Proposed method	Proposed method
		(Classic features)	(Combinational feature space)
MLP	67.44%	71.08%	98.74%

According to the results reported by Shen et al. and our findings, It’s obvious that Nonlinear and Time-Frequency processing methods excels at classic processing methods. Moreover, in comparison with our former study [22], it has been investigated that an optimal combination of these processing methods (i.e., TF and Linear and Nonlinear) improves the separability in a dramatic way.

Finally, experimental results show that there are significant information in HRV signal which can be extracted by the proposed method and used for prediction of SCD although there is no significant difference between normal ECG and those ones which are prone to SCD. We investigated that the 2 minutes interval before SCD can be used to distinguish between a person who is prone to SCD and a normal ECG. Also, the third minute interval before SCD possesses information indicating high risk of SCD that can be estimated through the proposed method. Moreover, as the time approaches to SCD, the risk of SCD increases which is expectable from the medical perspective. In reviewing the forth minute before SCD, the risk of SCD exists yet although it has been decreased in comparison with the previous intervals which are closer to the SCD. Generally, healthy and unhealthy persons can be classified by detecting heart attack and tachyarrhythmia before SCD, because patients who show signs of a previous heart attack or having the hard tachyarrhythmia are susceptible for SCD, and finally they catch SCD. It is noticeable that by getting closer to the SCD from the forth interval, the percentage of correct detection of SCD rise dramatically and then climb sharply for the closer intervals to the SCD. Moreover, considering the obtained results, it could be seen that MLP classifier has better performance in detection of SCD than k-NN classifier. Finally, our findings about detection of SCD can help doctors and Treatment centers to be aware of SCD even 4 minutes before happening to prevent incident and do something that save the life of unhealthy person. Since features of the Wigner-Ville transformed HRVs are a comprehensive form of frequency features which Time-Domain Information has been added to them, it seems simultaneous use of Time and Frequency information through TF analysis includes useful information which can’t be available using Classic (i.e., Time or Frequency) methods. Also, it seems the usage of Nonlinear methods can bring some more information in comparison with the common Linear methods due to the Nonlinear nature of HRV signal. So, Nonlinear methods can be considered as supplementary to improve classic (i.e., Time or Frequency) methods.

Finally, it is reminded that to investigate the dependence of samples, we did all experiments two times: 1) using all channels and 2) using only one channel per patient. The obtained results revealed no significant difference between them. In other words, the classification performance (Accuracy) of classifiers were really close to each other. On the other hand, the accuracy of k-NN classifier (as a non-parametric classifier) was close to MLP, which indicates the reliability of results. It is noticeable that we could never claim that channels from same patients are independent, and having a small number of observations compared to the total number of features was the main reason to use whole channels as input data, and then apply leave-one-out method to train classifiers.

In future studies, we intend to apply Nonlinear and Chaotic processings to ECG signals to extract new features, and also use other classifiers to improve the accuracy of prediction in much more time before SCD.
